# Five novel pathogenic *FZD4* variants identified in familial exudative vitreoretinopathy

**DOI:** 10.1016/j.aopr.2025.07.003

**Published:** 2025-07-25

**Authors:** You Wang, Qiong Wang, Limei Chen, Tao Cai, Xiaoyan Ding

**Affiliations:** a Department of Ophthalmology, Sichuan Provincial People's Hospital, University of Electronic Science and Technology of China, Chengdu, China; b State Key Laboratory of Ophthalmology, Zhongshan Ophthalmic Center, Sun Yat-sen University, Guangdong Provincial Key Laboratory of Ophthalmology and Visual Science, Guangzhou, China

**Keywords:** Familial exudative vitreoretinopathy, *FZD4*, Norrin/β-catenin, Genotype, Phenotype

## Abstract

**Purpose:**

Familial exudative vitreoretinopathy (FEVR) is a genetically heterogeneous retinal vascular disorder, with nearly half of the cases attributed to mutations in genes involved in the Norrin/β-catenin signaling pathway. This study aimed to identify and functionally characterize novel *FZD4* variants in patients with FEVR.

**Methods:**

Genetic sequencing of *FZD4* was performed in a cohort of FEVR families, leading to the identification of five novel variants: c.434G ​> ​A, c.610T ​> ​G, c.844T ​> ​C, c.277C ​> ​T, and c.1155delC. Bioinformatic predictions, comprehensive clinical evaluations, and dual-luciferase reporter assays were conducted to assess the functional impact and pathogenicity of these variants.

**Results:**

All five *FZD4* variants were found to significantly reduce β-catenin signaling activity compared to wild-type *FZD4*. Among them, two variants previously classified as variants of uncertain significance (VUS) demonstrated functional impairment and clinical segregation consistent with pathogenicity, supporting their reclassification as disease-causing mutations.

**Conclusions:**

These findings expand the known mutational spectrum of *FZD4* in FEVR and highlight the critical role of functional validation in the interpretation of novel and uncertain variants. Incorporating experimental assays can improve diagnostic accuracy and inform clinical genetic counseling.

## 1 Introduction

Familial exudative vitreoretinopathy (FEVR) is a hereditary retinal disorder primarily characterized by incomplete vascularization of the peripheral retina at birth.^1^ This condition can lead to various complications, including retinal neovascularization and detachment.^2^ With advancements in genetic sequencing technologies, at least 25 genes have been identified as linked to FEVR.^3^ The most extensively studied genes are those involved in the Norrin/β-catenin signaling pathway, including *NDP*, *FZD4*, *LRP5*, and *TSPAN12*.^4^ Mutations in these genes reduce β-catenin nuclear accumulation and inhibit the transcription of downstream targets such as *c-MYC* and *SOX9*.^5^^,^^6^

*FZD4* is one of the most frequently mutated genes, accounting for approximately 6%–27% of FEVR cases.^3^ Located on chromosome 11q14.2, the *FZD4* gene consists of two exons that encode a protein of 537 amino acids.^7^ The FZD4 protein contains several essential structural domains, including an N-terminal extracellular cysteine-rich domain (CRD), seven transmembrane helices, and a C-terminal intracellular domain. The CRD is the key region that binds Norrin, a cysteine-rich secreted protein serving as a ligand in the Wnt pathway.^8^ The intracellular domains, including the K-T/S-XXX-W motif, the PDZ-binding motif, and the S/T-X-V PDZ recognition motif, are involved in the recruitment of Dishevelled 2.^9^^,^^10^ In summary, the Norrin-FZD4 complex activates the β-catenin-dependent canonical Wnt signaling pathway in a manner dependent on LRP5, playing a crucial role in the development of retinal vasculature.^5^ To date, over 100 *FZD4* variants have been reported; however, most have not been functionally validated in the context of the signaling pathway.^11^, ^12^, ^13^ The absence of proper validation may lead to the misclassification or oversight of specific pathogenic mutations that are currently categorized as variants of uncertain significance (VUS). Functional assays targeting the Norrin/β-catenin pathway are essential for determining the pathogenicity of VUS and for advancing our understanding of disease-causing mutations.

In this study, we reported five novel pathogenic mutations in the *FZD4* gene in six unrelated families. To evaluate their functional impact, we also conducted Dual-luciferase reporter assays to assess their effects on the Norrin/β-catenin signaling pathway.

## 2 Methods

### 2.1 Patients

This study adhered to the principles of the Declaration of Helsinki and was approved by the review boards of Sichuan Provincial People's Hospital. All participants or their guardians were fully informed and engaged in the process, providing their consent after a thorough and transparent explanation.

This study involved six individuals diagnosed with FEVR and their family members. The clinical diagnosis of FEVR was based on characteristic abnormalities in the peripheral retinal vasculature and associated complications, including peripheral retinal vascular underdevelopment, retinal neovascularization, retinal folds, and retinal detachment. Patients with suspected retinopathy of prematurity were excluded. All participants underwent comprehensive, age-appropriate ophthalmic examinations, including best-corrected visual acuity (BCVA), measurement of intraocular pressure (IOP), fundus examination, ocular ultrasonography, fundus photography, fundus fluorescein angiography (FFA), and optical coherence tomography (OCT).^14^ The severity levels of the subjects were assessed using Trese's classification system, providing a detailed evaluation of their conditions.^15^ Pathogenic myopia is characterized by an ocular axis exceeding 26 ​mm, along with at least one pathological alteration in the fundus, including posterior scleral staphyloma, myopic macular degeneration, choroidal retinal atrophy, choroidal neovascularization, and macular hole.^16^

### 2.2 Genetic testing

Genomic DNA extraction was performed following the protocol described in prior research.^17^ Whole-exome sequencing (WES) was performed on the probands in this cohort. The identified variants were validated via Sanger sequencing across family members. Reported variants were excluded by querying databases such as HGMD, dbSNP 151, and gnomAD (http://www.gnomad-sg.org), as well as reviewing the relevant literature. In silico pathogenicity assessment of missense variants incorporates tools including SIFT (http://provean.jcvi.org/index.php), PolyPhen-2 (http://genetics.bwh.harvard.edu/pph2/), Mutation Taster (http://www.mutationtaster.org), Alpha Missense (https://alphamissense.hegelab.org/search), PROVEAN (http://provean.jcvi.org/index.php), and CADD (https://cadd.gs.washington.edu), complemented by conservation analysis.^18^ Sequence variant classification adhered to the criteria outlined in the ACMG standards and guidelines.^19^

### 2.3 Plasmid construction

The wild-type sequences *TSPAN12*, *FZD4*, and *LRP5*, along with five mutant *FZD4* sequences (c.277C ​> ​T, c.434G ​> ​A, c.610T ​> ​G, c.844T ​> ​C, c.1155delC) and the PGL4.1-Renilla sequence were subcloned into the pcDNA 3.1 plasmid vector containing CMV, T7 promoters, and a FLAG tag. All plasmids were confirmed through sequencing. The plasmid DNA for transfections was prepared as mentioned in our previous study.^14^

### 2.4 Dual-luciferase assays

To evaluate the impacts of *FZD4* variants on Norrin/β-catenin signaling activity, HEK 293STF cells (which is from Pro. Zhu of Sichuan Provincial People's Hospital) were seeded onto a 24-well plate, and co-transfected with the plasmids mix (100 ​ng *TSPAN12*, 100 ​ng *LRP5*, and 100 ​ng pGL4-Renilla luciferase control plasmid) and 100 ​ng wild type (WT) or mutant *FZD4*. The transfection reagents included Lipofectamine 3000, P3000, and Opti-MEM I (ThermoFisher). After being cultured for 36 ​h, cells were activated with 50 ​ng of Norrin (R&D Systems), which harvested 12 ​h later for luciferase assays. The detailed procedures were also described in previous studies.^14^

### 2.5 Statistical analysis

In this study, one-way analysis of variance was employed to evaluate the overall differences among multiple groups. When the ANOVA results showed significant intergroup differences (*P* ​< ​0.05), Dunnett's test was used to compare multiple experimental groups against a common control group. All statistical analyses were performed using SPSS 22.0 for Windows (IBM Corporation, Armonk, NY, USA). Statistical significance was determined based on a 95% confidence interval, with *P* ​< ​0.05 considered statistically significant.

## 3 Results

### 3.1 Genetic findings

**Fig. 1:**
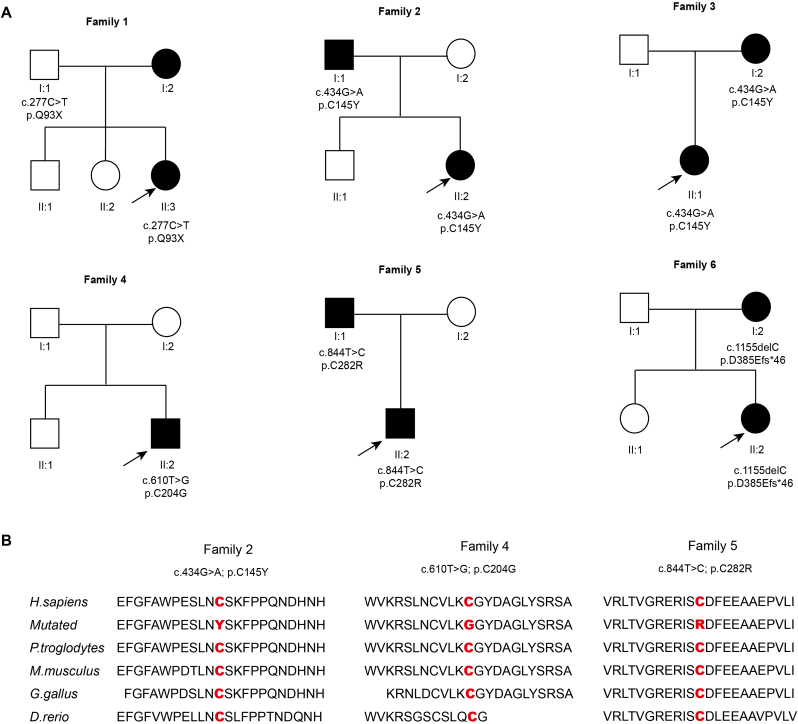
The six FEVR family pedigrees with variants in *FZD4* and the conservation analysis of three missense variants. (A) Six pedigrees of the FEVR family are presented. Patients are represented in black, and the probands are indicated by black arrows. (B) The conservation analysis of three missense variants indicated that these amino acids are highly conserved.

**Fig. 2:**
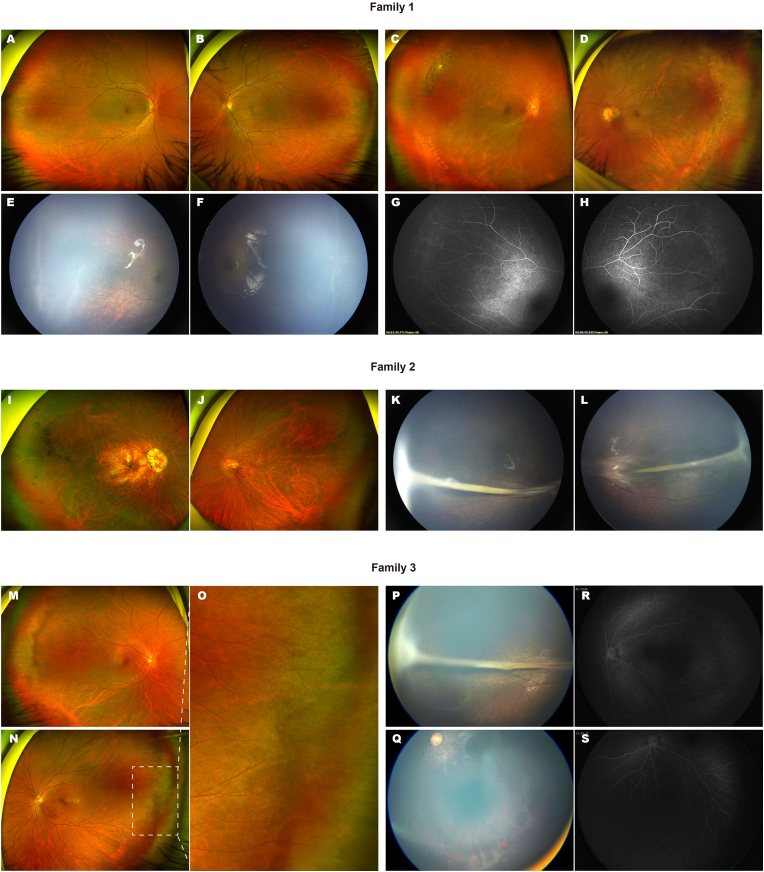
The schematic diagram displayed illustrates the structure of the FZD4 protein along with the locations of mutation sites. The red dots represent the mutation sites identified in this study. The red text highlights novel variants that are being reported here for the first time, while the black text indicates previously documented mutations.

The WES was performed on FEVR probands; among them, five novel *FZD4* variants were identified in six probands and subsequently validated using Sanger sequencing in family members. These probands and family members were enrolled in the present study. All subjects were of Han Chinese ethnicity. The five novel variants are shown in Table 1, included three missense mutations (c.434G ​> ​A, p.C145Y; c.610T ​> ​G, p.C204G; c.844T ​> ​C, p.C282R), one nonsense mutation (c.277C ​> ​T, p.Q93X), and one frameshift mutation (c.1155delC, p.D385Efs46∗). None of the five mutations were found in the gnomAD (version 2.1) control database. The c.610T ​*>* ​G mutation was *de novo*. The remaining four variants showed co-segregation with disease within their respective families (Fig. 1A). Conservation analysis revealed that the three missense mutations affected highly conserved amino acid residues (Fig. 1B). In the initial bioinformatic classification, p.C145Y and p.C282R were categorized as VUS. The p.Q93X and p.C145Y mutations are located in the CRD domain of the FZD4 protein. Amino acid positions 145, 204, and 282 have been reported to contain various mutations (Fig. 2).

**Table 1:** Causative mutations in the five families.

Family number	Nucleotide change	Protein change	Position	Mutation Type	Co-segregation	SIFT	Poly-Phen 2	Mutation Taster	Alpha Missense	PROVEAN (Score)	CADD	Functional significance
1	c.277C ​> ​T	p.Q93X	chr11:86665851	Nonsense	Maternal	–	–	Deleterious		–	42	Pathogenic
3	c.434G ​> ​A	p.C145Y	chr11:86663364	Missense	Maternal	Damaging (0.001)	Probably damaging	Deleterious	Likely pathogenic	Deleterious (−10.23)	33	Pathogenic
4	c.610T ​> ​G	p.C204G	chr11:86663188	Missense	*De novo*	Damaging (0.022)	Probably damaging	Deleterious	Likely pathogenic	Deleterious (−9.59)	26.4	Pathogenic
5	c.844T ​> ​C	p.C282R	chr11:86662954	Missense	Paternal	Damaging (0.00)	Probably damaging	Deleterious	Likely pathogenic	Deleterious (−11.21)	31	Pathogenic
6	c.1155delC	p.D385Efs∗46	chr11: 86662643	Frameshift	Maternal	–	–	–	–	–	–	Pathogenic

### 3.2 Clinical findings

**Fig. 3:**
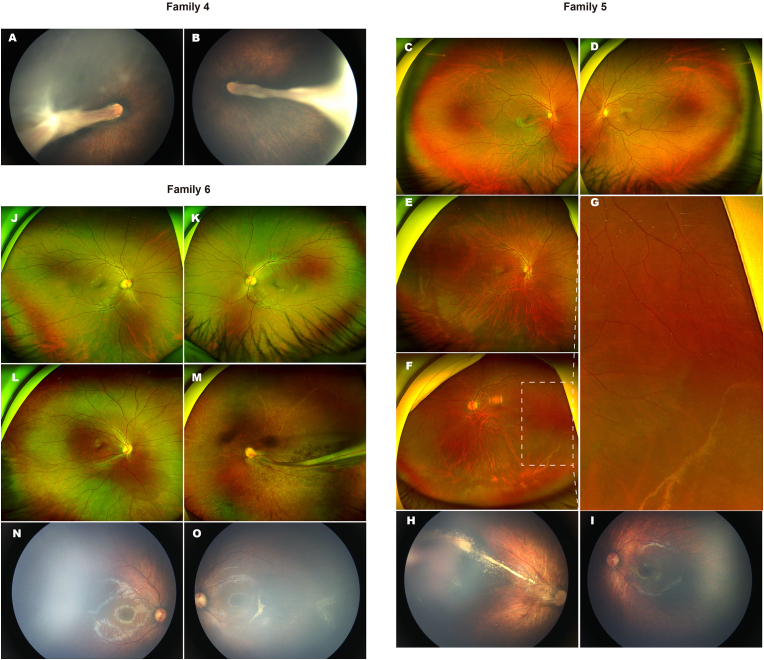
Retinal features of affected individuals from Families 1–3. **Family 1:** (A, B) Fundus images of the proband's father showing normal retinal vasculature. (C, D) The proband's mother shows peripheral avascular zones and lattice degeneration in both eyes. (E, F) Fundus photographs of the proband demonstrate peripheral ridge-like changes (G, H) Fluorescein angiography of the proband reveals peripheral non-perfusion. **Family 2:** (I, J) Fundus images of the proband's father reveal peripheral vascular underdevelopment in both eyes and pathogenic myopic changes in the right eye. (K, L) The proband presents with bilateral retinal folds. **Family 3:** (M, N) The proband's mother shows bilateral peripheral vascular underdevelopment. (O) Magnified view of the peripheral retina in the mother's left eye. (P, Q) The proband demonstrates peripheral vascular abnormalities in the right eye and peripheral chorioretinal atrophy in the left eye.(R, S) Fluorescein angiography of the proband's left eye reveals non-perfusion in the temporal and inferior periphery.

**Fig. 4:**
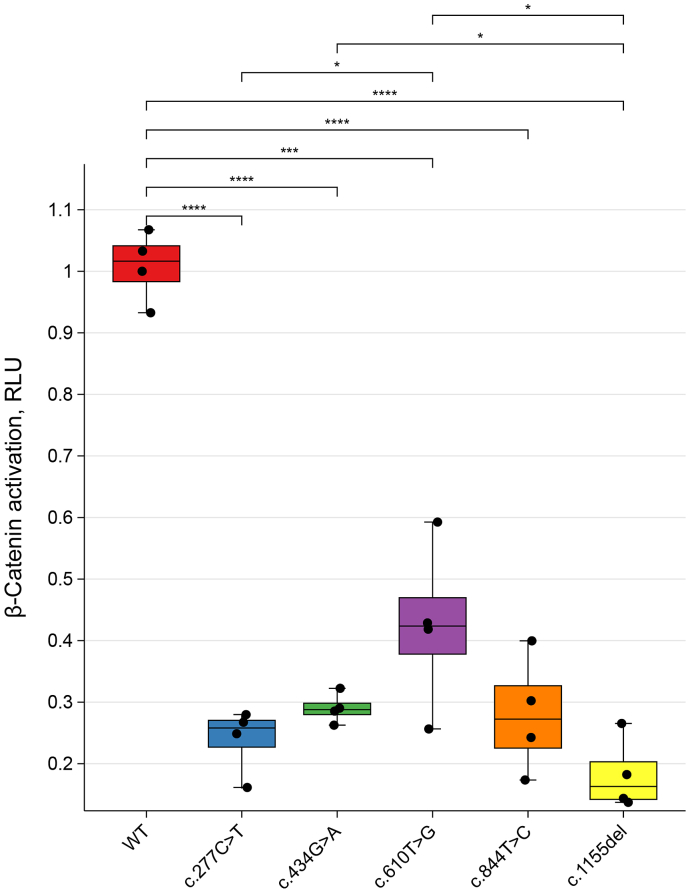
Retinal features of affected individuals from Families 4–6. **Family 4:** (A, B) The proband presents with bilateral retinal folds. **Family 5:** (C, D) Fundus images of the proband's mother show normal retinal vasculature in both eyes. (E, F) The proband's father exhibits peripheral retinal vascular underdevelopment in both eyes. (G) Magnified view of the temporal retina in the father's left eye shows straightened and underdeveloped peripheral vessels. (H, I) Fundus images of the proband reveal macular ectopia and peripheral neovascular lesions with extensive yellow-white exudates in the right eye, and peripheral vascular underdevelopment in the left eye. **Family 6:** (J, K) Fundus images of the proband's father show normal retinal findings. (L, M) The proband's mother demonstrates abnormal vascular development in the right eye and retinal folds in the left eye. (N, O) The proband shows a V-shaped configuration of temporal retinal vessels in both eyes.

A total of six probands and five affected family members from six unrelated families were included in the clinical analysis. Detailed clinical features are presented in Table 2. Among the probands, five were diagnosed before the age of one year, and one was diagnosed at three years of age. Of the 12 eyes from the six probands, six exhibited incomplete development of the peripheral retinal vasculature, five showed retinal folds, and one eye presented with peripheral retinal neovascularization along with macular ectopia. Of the ten eyes belonging to the five affected family members, nine showed signs of underdeveloped peripheral vascularization, including one eye that showed pathogenic myopia. Additionally, one eye exhibited retinal folds. Eyes with peripheral vascular underdevelopment generally retained good visual function, with an average BCVA of 0.122 ​± ​0.04 (logMAR). Representative fundus photographs of the probands and affected family members are shown in Fig. 3, Fig. 4.

**Table 2:** Clinical information of the patients and their family members.

Families	Family members	Age (Year)	Gender	Status	BCVA (log MAR)		Fundus features		Stages	
					OD	OS	OD	OS	OD	OS
1	I:1	39	F	UA	0.1	0.1	Normal	Normal	–	–
	I:2	28	M	A	0.1	0.1	Incomplete development of the peripheral retinal vasculature	Incomplete development of the peripheral retinal vasculature	1	1
	II:3^a^	1	M	A	NA	NA	Incomplete development of the peripheral retinal vasculature	Incomplete development of the peripheral retinal vasculature	1	1
2	I:1	33	M	A	0.2	0.1	Incomplete development of the peripheral retinal vasculature, posterior scleral staphyloma, choroidal atrophy	Incomplete development of the peripheral retinal vasculature	1	1
	I:2	37	F	UA	0.1	0.1	Normal	Normal	–	–
	II:2^a^	1	F	A	NA	NA	Retinal folds	Retinal folds	3	3
3	I:1	29	F	UA	0.1	0.1	Normal	Normal	–	–
	I:2	27	M	A	0.1	0.1	Incomplete development of the peripheral retinal vasculature	Incomplete development of the peripheral retinal vasculature	1	1
	II:1^a^	3	F	A	NA	NA	Retinal folds	Incomplete development of the peripheral retinal vasculature, choroidal atrophy	3	1
4	I:1	31	M	UA	0.1	0.1	Normal	Normal	–	–
	I:2	31	F	UA	0.1	0.1	Normal	Normal	–	–
	II:1	4	M	UA	NA	NA	Normal	Normal	–	–
	II:2^a^	1	M	A	NA	NA	Retinal folds	Retinal folds	3	3
5	I:1	42	M	A	0.1	0.1	Incomplete development of the peripheral retinal vasculature	Incomplete development of the peripheral retinal vasculature	1	1
	I:2	28	F	UA	0.1	0.1	Normal	Normal	–	–
	II:1^a^	1	M	A	NA	NA	macular ectopia, retinal neovascularization	Incomplete development of the peripheral retinal vasculature	3	1
5	I:1	32	M	UA	0.1	0.1	Normal	Normal	–	–
	I:2	31	F	A	0.2	1.3	Incomplete development of the peripheral retinal vasculature	Retinal folds	1	3
	II:1	3	F	UA	NA	NA	Normal	Normal	–	–
	II:2^a^	1	F	A	NA	NA	Incomplete development of the peripheral retinal vasculature	Incomplete development of the peripheral retinal vasculature	1	1

OD: Oculus Dexter; OS: Oculus Sinister; M: Male; F: Famale; A: affected; UA: unaffected; NA: none available; BCVA: best-corrected visual acuity.

a Proband.

### 3.3 Dual-luciferase assays

**Fig. 5:**
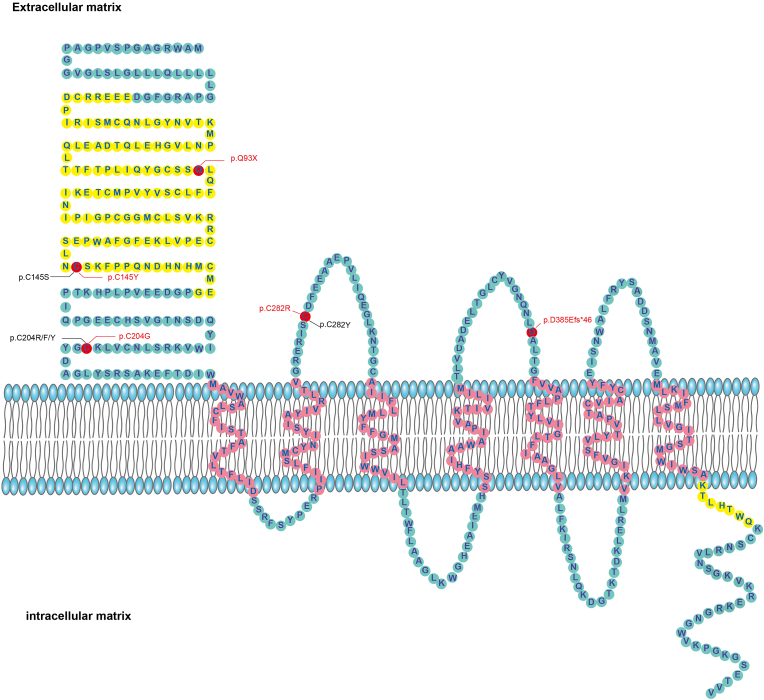
Impaired activation of the Norrin/β-catenin signaling pathway by five *FZD4* variants compared with wild-type. Each condition was tested in quadruplicate wells and independently repeated three times. Error bars represent standard deviation. *N* ​= ​4. ∗∗∗∗*P* ​< ​0.0001. RLU, relative luciferase units.

To evaluate the impact of the five *FZD4* mutations on the Norrin/β-catenin signaling pathway, we performed dual-luciferase reporter assays. The value of Firefly/Renilla showed that all five mutant *FZD4* plasmids significantly reduced β-catenin signaling activity compared to the WT, with reductions ranging from 50% to 90% (Fig. 5). Based on these functional results in vitro assays, combined with the established pathogenicity of other missense variants at the same positions (Fig. 2), and the absence of these variants in population databases such as gnomAD, both p.C145Y and p.C282R were also reclassified as pathogenic.

## 4 Discussion

Previous studies have established that the *FZD4* gene promotes retinal vascular development by activating the Norrin/β-catenin signaling,^20^ and numerous *FZD4* mutations have been identified in patients with familial exudative vitreoretinopathy (FEVR).^4^^,^^21^^,^^22^ In this study, we identified five novel *FZD4* variants (c.434G ​> ​A, c.610T ​> ​G, c.844T ​> ​C, c.277C ​> ​T, and c.1155delC), all of which were ultimately classified as pathogenic based on the classification guidelines of sequence variants.

Dai et al. previously reported that mutations involving cysteine residues responsible for forming intramolecular disulfide bonds often lead to abnormal protein folding or maturation of FZD4.^10^ Specifically, they found that p.C145S and p.C204R significantly attenuate β-catenin signaling activity, which is consistent with our findings that p.C145Y and p.C204G also reduce β-catenin pathway activation. In the wild-type FZD4 protein, cysteine residues at positions 121 and 145, as well as 204 and 282, form intramolecular disulfide bonds that are critical for maintaining proper protein conformation.^10^ Disruption of these bonds likely impairs the tertiary structure of the protein, thereby compromising its function. These results highlight the potential pathogenicity of mutations at functionally critical residues. Additionally, the nonsense mutation p.Q93X and the frameshift mutation p.D385Efs46∗ are predicted to cause premature termination of translation, leading to truncated, non-functional FZD4 protein products. Collectively, these findings expand the known mutational spectrum of *FZD4* and provide further insight into the molecular mechanisms underlying FEVR.

In silico prediction tools like SIFT, PolyPhen-2, PROVEAN, and CADD can offer initial insights into the potential pathogenicity of genetic variants. However, these tools are limited by the assumptions of their algorithms and the datasets used for training, which may lead to inconsistent or inconclusive results, particularly for rare or novel mutations.^23^^,^^24^ Functional validation assays, by contrast, provide direct biological evidence of a variant's effect on gene function and signaling activity. In this study, we employed a dual-luciferase reporter assay to evaluate the impact of five novel *FZD4* mutations on Norrin/β-catenin signaling. Among them, p.C145Y and p.C282R were initially classified as VUS. However, both demonstrated a significant reduction in pathway activity, consistent with other pathogenic *FZD4* mutations. Together with supporting evidence from structural biology and prior reports of similar amino acid substitutions at the same residues, these findings justify their reclassification as pathogenic. Thereby, the accurate classification of VUS is crucial in clinical practice, particularly for conditions such as FEVR, where early diagnosis facilitates timely intervention. Misclassification may lead to either overdiagnosis or missed opportunities for genetic counseling and preventive care. Our results emphasize the indispensable role of functional assays in bridging the gap between genetic findings and clinical interpretation. Incorporating experimental validation into the variant classification workflow not only improves diagnostic precision but also enhances our mechanistic understanding of disease pathogenesis.

It is important to recognize that FEVR is a clinically heterogeneous disorder, with manifestations ranging from asymptomatic peripheral vascular anomalies to severe, vision-threatening complications such as retinal detachment. In this study, probands often presented with more severe phenotypes than their affected family members. While this may imply intrafamilial variability, the observation is likely influenced by ascertainment bias: individuals with mild or no symptoms, particularly if they are children, or if they retain good visual acuity, may remain undiagnosed and are thus less likely to undergo clinical evaluation. Consequently, those with advanced or symptomatic disease are more likely to come to medical attention, creating an apparent generational disparity in disease severity.

The suppression of the Norrin/β-catenin pathway in vitro also did not always align with clinical severity. For instance, the c.610T ​> ​G variant had a milder effect on β-catenin signaling than c.277C ​> ​T, yet was linked to a more severe phenotype. These findings suggest that additional genetic modifiers, epigenetic regulation, or environmental factors may influence disease progression, complicating the relationship between genotype and phenotype.^25^ Such variability also underscores the need for caution when interpreting familial segregation patterns. Mildly affected individuals, especially those with only peripheral vascular anomalies, should not be overlooked, as their presence can provide crucial support for pathogenic variant classification.

Several limitations should be acknowledged in this study. First, the sample size is relatively small, which may restrict the generalizability of our findings across the broader FEVR population. As FEVR exhibits considerable phenotypic and genotypic heterogeneity, larger cohorts are essential to validate the clinical relevance of the novel *FZD4* mutations identified and to establish clearer genotype–phenotype correlations. Second, although luciferase reporter assays provide a reliable method to evaluate the functional impact of *FZD4* variants on the Norrin/β-catenin pathway, they do not fully recapitulate the complex in vivo retinal microenvironment. Third, because this is a retrospective observational study conducted at a tertiary referral center, there may be an inherent selection bias toward more severe or symptomatic cases. This could skew the perceived phenotypic severity and mutation penetrance.

## 5 Conclusions

In this study, we found that *FZD4* mutations accounted for 14.67% of cases in our large FEVR cohort. Through comprehensive bioinformatic prediction, clinical phenotyping, and in vitro functional validation, we identified five novel pathogenic variants in the *FZD4* gene. This study expands the mutations identified in the *FZD4* gene associated with FEVR and emphasizes the need for functional assays to evaluate uncertain variants.

## Study approval

The authors confirm that any aspect of the work covered in this manuscript that involved human patients was conducted with the ethical approval of all relevant bodies and the study was performed in accordance with the Declaration of Helsinki,and the protocol was approved by the Ethics Committee of Sichuan Provincial People's Hospital (LS-2023-046).

## Author contributions

The authors confirm contribution to the paper as follows: Conception and design of study: YW, XD; Data collection: YW, QW, LC; Analysis and interpretation of results: QW, YW, TC; Drafting the manuscript: YW, XD; All authors reviewed the results and approved the final version of the manuscript.

## Funding

This work was supported by the 10.13039/501100001809National Natural Science Foundation of China (82271092).

## Declaration of competing interest

The authors declare that they have no known competing financial interests or personal relationships that could have appeared to influence the work reported in this paper.

## Abbreviations

FEVR: familial exudative vitreoretinopathy
VUS: variant of uncertain significance
BCVA: best-corrected visual acuity
OCT: optical coherence tomography
FFA: fundus fluorescein angiography
IOP: intraocular pressure
